# Glutathione-linked antioxidant defence limits *Edwardsiella tarda*-induced cytotoxicity in organic acid-treated gill epithelial cells

**DOI:** 10.3389/fcimb.2026.2008102

**Published:** 2026-10-26

**Authors:** Igori Balta, Ioan Pet, Lavinia Stef, Ozan Gundogdu, Florica Morariu, Elena Pet, Iuliana Cretescu, Ducu Stef, Nicolae Corcionivoschi

**Affiliations:** 1 Faculty of Bioengineering of Animal Resources, University of Life Sciences King Mihai I from Timisoara, Timisoara, Romania; 2 Department of Infection Biology, Faculty of Infectious & Tropical Diseases, London School of Hygiene & Tropical Medicine, London, United Kingdom; 3 Faculty of Management and Rural Tourism, University of Life Sciences King Mihai I from Timisoara, Timisoara, Romania; 4 Centre for Modeling Biological Systems and Data Analysis “Victor Babes”, University of Medicine and Pharmacy, Timișoara, Romania; 5 Faculty of Food Engineering, University of Life Sciences King Mihai I from Timisoara, Timisoara, Romania; 6 Veterinary Sciences Division, Agri-Food and Biosciences Institute, Belfast, Northern Ireland, United Kingdom; 7 Academy of Romanian Scientists, Bucharest, Romania

**Keywords:** AuraAqua (Aq), barrier integrity, cytotoxicity, *Edwardsiella tarda*, oxidative stress, RTgill-W1 cell line

## Abstract

Cytotoxic injury to gill epithelial cells is a major consequence of pathogen infection and can compromise barrier function in aquaculture species. *Edwardsiella tarda* is an important bacterial pathogen in aquaculture. Infection of gill epithelial cells can damage barrier integrity, induce cytotoxicity, and trigger oxidative and inflammatory stress responses. This study evaluated whether AuraAqua (Aq), an organic acid-based antimicrobial mixture, protects RTgill-W1 cells from *E. tarda*-induced cytotoxicity and the associated inflammatory and oxidative stress responses. Aq showed a minimum inhibitory concentration of 1% and a minimum bactericidal concentration of 2%. Aq treatment reduced *E. tarda* adhesion and intracellular bacterial recovery, indicating impaired early host-pathogen interactions. Importantly, Aq was not directly cytotoxic to RTgill-W1 cells at the tested concentrations (0, 0.1, 0.2, 0.5, 1 and 2%) and reduced infection-associated LDH release while preserving cell viability and transepithelial electrical resistance. These outcomes were accompanied by increased expression of tight-junction-associated genes, including *cldn-10e*, *cldn-30*, and *zo-1*. During infection, Aq enhanced intracellular glutathione (GSH) levels, reduced superoxide dismutase (SOD) and catalase (CAT) activity, decreased H_2_O_2_ accumulation, and decreased NF-κB p65 activation. Aq also significantly downregulated IL-8, IL-1β, and TNF-α expression in infected cells. Together, these findings show that Aq can stimulate increased endogenous GSH production, which primarily protects RTgill-W1 cells and limits *E. tarda*-induced cytotoxic injury. This strengthens antioxidant defence, maintains epithelial barrier integrity, and suppresses NF-κB-associated inflammatory signalling.

## 1 Introduction

Fish gills reflect a biological compromise: a thin, highly exposed epithelium that must sustain gas exchange and ion regulation while defending against waterborne microorganisms (Sackville et al., 2024). This architecture places epithelial barrier integrity at the centre of early host–pathogen interactions. The rainbow trout RTgill-W1 cell line provides a tractable model of this interface because it forms electrically resistive monolayers, expresses tight-junction-associated genes and mounts inducible innate responses (Trubitt et al., 2015; Misk et al., 2022). In RTgill-W1 monolayers, transepithelial electrical resistance (TEER) provides a functional readout of barrier behaviour, while *cldn-10e*, *cldn-30* and *zo-1* are established tight-junction-associated transcripts in this model (Trubitt et al., 2015). RTgill-W1 cells can also undergo pathogen-responsive transcription: FliC from *E. tarda* elicits a distinct innate-response programme, and challenge with *Piscirickettsia salmonis* produces an early immune-like response in the same epithelial background, with intracellular bacteria detectable during infection (Valdebenito et al., 2025).

Oxidative-stress adaptation is metabolically integrated with intracellular proliferation, identifying a large H_2_O_2_-responsive proteomic network and metabolic determinants required for survival and dissemination (Wang and Sun, 2023). Reactive oxygen species, particularly H_2_O_2_, function as regulated signalling intermediates at physiological concentrations but become damaging when production exceeds local buffering and repair capacity (Sies and Jones, 2020). In RTgill-W1 cells, experimentally induced mitochondrial oxidative stress increases ROS and is accompanied by depletion of glutathione (GSH) and catalase, demonstrating that this cell line is sensitive to changes in intracellular redox buffering (Solhaug et al., 2023). Dietary GSH mitigates hypoxia-associated structural and oxidative injury in grass-carp gills, while dietary malic/citric-acid combinations in gibel carp alter GSH status together with physiological and immune indices (Zhang et al., 2020; Zeng et al., 2025a). Moreover, GSH dietary supplementation has been shown to enhance growth performance, survival, antioxidant capacity, and non-specific immunity in common carp exposed to ammonia, thereby alleviating the adverse effects of ammonia stress. GSH reduced inflammation in gill and liver tissues by modulating the expression of antioxidant-, inflammatory-, and stress-related genes, including SOD, CAT, GSH-Px, IL-1β, IL-6, IL-8, IL-10, IFN-γ, TNF-α, Hsp70, and Hsp90. These findings indicate that GSH can protect common carp against ammonia-induced toxicity (Xue et al., 2022). Redox state also provides a plausible bridge from epithelial stress to inflammatory output. H_2_O_2_ and other oxidant signals can modify pathways that converge on NF-κB, but the direction and biological consequence of that interaction are context-dependent (Qin et al., 2024). Fish-specific evidence is now available: in *Aeromonas hydrophila* infection models, GSH or taurine/GSH treatment reduced ROS accumulation, restrained NF-κB activation and altered IL-1β and TNF-α expression, linking redox buffering to inflammatory control without making GSH synonymous with NF-κB inhibition (Li et al., 2026). *E. tarda* infection of flounder gill cells likewise engages NF-κB-associated signalling and inflammatory cytokines during infection-induced cell death (Sun and Jiang, 2026).

*Edwardsiella tarda* remains an important bacterial pathogen in aquaculture because of its capacity for environmental persistence, systemic infection, intracellular survival, immune evasion and disease across diverse fish hosts (Xu and Zhang, 2014; Leung et al., 2019; Aktar et al., 2026). Yet its economic importance is much less securely quantified than its pathogenic importance. The central reason is not merely a shortage of farm-accounting studies; it is also taxonomic. The economic burden attributable specifically to *E. tarda sensu stricto* remains poorly resolved, partly because historical disease records predate the taxonomic separation of fish-associated *Edwardsiella*. The scale of the broader problem is nevertheless substantial: Kumar et al. (2024) estimated annual direct losses of USD 5.2–17.6 million and a total negative economic impact of USD 15.5–45.9 million from edwardsiellosis in the U.S. catfish industry (Kumar et al., 2024). For perspective, against the USD 437 million in U.S. catfish sales reported for 2023 (USDA National Agricultural Statistics Service, 2024), these modelled values correspond to approximately 1.2–4.0% of annual sales for direct losses and 3.5–10.5% for total economic impact. These estimates illustrate the production-level consequences that pathogenic *Edwardsiella* spp. can impose on intensive aquaculture.

A mechanistic gap therefore remains at the intersection of *E. tarda* pathogenesis and gill epithelial protection. It is unknown whether sub-inhibitory exposure to an organic-acid-based antimicrobial mixture can interfere with early *E. tarda*–epithelial interactions while preserving barrier function and constraining the redox–inflammatory disturbances generated during infection. Fish studies show that dietary organic-acid formulations can alter disease outcomes, while malic/citric-acid supplementation can influence redox and immune indices; neither observation establishes a mechanism shared by all acids or formulations (Rømer Villumsen et al., 2020; Zhang et al., 2020; Liliana et al., 2024). More mechanistic work with organic-acid mixtures has begun to separate pathogen-facing from host-facing effects. In tilapia gut models, Aq reduced *Streptococcus agalactiae* infection together with H_2_O_2_ and inflammatory readouts; in shrimp epithelial cells, the formulation attenuated infection-associated H_2_O_2_ and NF-κB p65 activation during *Enterocytozoon hepatopenaei* challenge (Liliana et al., 2024). A study using *Aeromonas hydrophila* in tilapia and shrimp primary gut cells found that epithelial barrier integrity was preserved, as indicated by maintained transepithelial electrical resistance (TEER). Furthermore, Aq treatment reduced measures of cytotoxicity and oxidative stress within the cells, suggesting that the antimicrobial mixture not only limits pathogen interaction but also supports cellular resilience against infection-related damage (Morariu et al., 2026).

Organic acids offer a promising strategy to improve aquaculture productivity while supporting more sustainable fish-farming practices. Including them in aquafeed may also improve feed efficiency and support better growth and overall fish health (Rasidi et al., 2024). When used in combination, they can enhance antioxidant status and support glutathione-related defence mechanisms in fish. As such, supplementing diets with combinations of citric acid, fumaric acid, and L-malic acid has been shown in largemouth bass to improve hepatic antioxidant capacity, including increased CAT, T-AOC, and GSH-PX activity, together with upregulated expression of antioxidant-related genes (Ma et al., 2026). Because GSH-PX activity depends on the availability and turnover of reduced glutathione, these findings suggest that organic acid mixtures may help promote glutathione metabolism and strengthen cellular protection against oxidative stress. Therefore, dietary strategies may represent a biologically relevant approach to stimulate GSH-associated antioxidant responses and improve hepatic resilience under stress conditions (Naiel et al., 2026). Their use as mixtures is justified in real biological systems by the several overlapping effects that occur simultaneously: antimicrobial pressure (Gómez-García et al., 2019), reduced bacterial adhesion or invasion (Amrutha et al., 2017), antioxidant support (Long et al., 2018), barrier protection (Yang et al., 2019), and inflammatory modulation (Bialkowski et al., 2023). This study aimed to use the RTgill-W1 gill epithelial cell line as an advanced *in vitro* model to evaluate the protective effect of AuraAqua (Aq), an organic acid-based antimicrobial mixture, against *E. tarda*-induced cellular injury. By focussing on cell viability, cytotoxicity, epithelial barrier integrity, oxidative stress modulation, glutathione-associated antioxidant defence, and inflammatory signalling, this study supports the development of future precision aquaculture strategies that use functional antimicrobial mixtures to strengthen gill epithelial resilience before pathogen-induced damage occurs. In the present study, AuraAqua combines citric acid, sodium citrate, malic acid, citrus extract, olive extract, and supporting carrier components, which may act together to reduce *E. tarda*–host cell interactions, enhance glutathione-associated antioxidant responses, and limit NF-κB-linked inflammation. This multi-component approach is therefore likely to provide more robust protection than a single organic acid, particularly in complex infection settings where bacterial virulence, oxidative stress, inflammatory signalling, and epithelial barrier disruption occur simultaneously.

## 2 Materials and methods

### 2.1 Cell lines, bacteria and organic acid mixture

RTgill-W1-CRL-2523 cell line was obtained from American Type Culture Collection (ATCC) and grown in Dulbecco’s modified Eagle medium (DMEM) (Thermo Fisher, UK) supplemented with 10% FBS (Thermo Fisher, UK) and 1% penicillin/streptomycin (Thermo Fisher, UK). The cells were cultured at 20 °C in a non-ventilated cell culture flask and sub-cultured 1:2 once every 10th day using trypLE (Thermo Fisher, UK). For experiments, cells were seeded (1 × 10^5^ cells) 15 days before the experiments and allowed to reach >80% confluence. The cell number reached full confluence on the day of the experiment. *Edwardsiella tarda* (*E. tarda* ATCC 15947) was grown in tryptic soy broth or agar (TSB; Thermo Fisher, UK), or DMEM at 28 °C. The AuraAqua (natural antimicrobial mixture) contains: 5% maltodextrin, 1% sodium chloride, 18% sodium citrate, 42% citric acid, 10% silica, 12% malic acid, 3% olive extract and 9% citrus extract (w/w) (Environtech, Dublin, Ireland). Bio-Science Nutrition Ireland supplied the raw materials. Experiments were carried out in triplicate.

### 2.2 Minimum inhibitory and minimum bactericidal concentration

We have used the two-fold tube dilution method to identify the concentrations at Aq inhibits bacterial growth (MIC), and induces bacterial death (MBC) (Marcu et al., 2026). Aq was diluted (8% to 0.015625% v/v) in TSB media. *E. tarda* was harvested by centrifugation, washed twice in PBS, resuspended in TSB media, and diluted to 1 × 10^5^ CFU/mL. Non-inoculated bijou (5 mL) tubes containing the same growth medium served as negative controls, while TSB tubes without Aq were inoculated with individual bacterial cultures as positive controls. *E. tarda* cultures were incubated at 28 °C for 24 h. Tubes with no visible growth were above the MIC. We took a 100 µL aliquot from each tube with no growth and inoculated it onto TSB agar plates. We repeated all assays in triplicate for each strain. The closest concentration to the determined MIC with no effect on survival was considered sub-inhibitory (sub-MIC, 0.5%).

### 2.3 Lactate dehydrogenase and cell viability measurement

The LDH measurement assessed the cytotoxic effects of Aq itself or following infection with *E. tarda* in the presence of 0.1, 0.2, 0.5, 1, and 2% Aq at 3-days post-infection. Cytotoxicity was determined by determining the LDH from RTgill-W1 cells upon infection. LDH levels were measured in the RTgill-W1 cell culture supernatants at 3-days post-infection using the cytotoxicity detection kit (Roche, Buckinghamshire, United Kingdom) according to the manufacturer’s guidelines. The measurements were performed at 500 nm using a spectrophotometer (FLUOstar Omega, Premier Scientific, Belfast, United Kingdom). Triplicate measurements were taken. Cell survival was evaluated by adding 10 μL of MTT reagent (0.5 mg/mL MTT) to each well and incubating for 3 h. This medium was then removed, and 100 μL of the solubilization solution was added to dissolve the MTT formazan. The plate was incubated overnight at 37 °C with 5% CO_2_. The absorbance of the MTT purple colour was measured on a multiwell plate reader (FLUOstar Omega, BMG Labtech, UK) using a 570 nm filter. Cell viability was expressed as a percentage of the control.

### 2.4 Infection assay

The RTgill-W1 cells and the *E. tarda* were prepared as described above. The RTgill-W1 cells were grown to a confluence of 80–90% (15 Days) using 6-well plates at pH 7.2. The *E. tarda* bacterium was added to MOI of 100 for cell infection (OD600 of ≈ 0.4) and the RTgill-W1 cells infected for 3 days (72h) in DMEM media supplemented with 0.1%, 0.2%, 0.5%, and 1% Aq. The pH during all the experimental infection studies was maintained at neutral values (pH 7.2). Bacterial adherence was performed by washing the infected RTgill-W1 cells in PBS and exposure to 0.1% Triton X100 in PBS for 15 min at 37 °C. Tenfold (10X) dilutions of the lysates were plated onto TSB agar at 28 °C for 24 hours and incubated for 2 days. To estimate the number of bacteria that invaded RTgill-W1 cells, the infected cells were washed in DMEM and then treated with gentamicin (400 μg/mL) in 2 mL DMEM (3 h at 28 °C) to inactivate (kill) the attached bacteria. The RTgill-W1 cells were lysed with 1 mL of 0.1% Triton X-100 in PBS for 15 min at 37 °C. Triplicate experiments were performed.

### 2.5 Measurements of H_2_O_2_, superoxide dismutase and catalase

The impact of Aq on SOD and CAT in *E. tarda*-infected RTgill-W1 cells was measured as previously described (Pet et al., 2025). The SOD (Thermo Fisher, Horsham, UK) and CAT (Abcam, Trumpington, UK, ab83464) levels were measured using commercially available kits as per manufacturer’s instructions. The SOD and CAT levels were measured in sonication-lysed infected cells for 60 s (4x) at 4 °C (on ice in 1% saline solution) and recovered by centrifugation at 2500 rpm for 5 min at 4 °C (Ultrawave DP200-00, Ultrawave Ltd., Cardiff, UK). Hydrogen peroxide (H_2_O_2_) in the infected RTgill-W1 cells was measured using a PeroxiDetect™ Kit (Sigma-Aldrich, Gillingham, UK). All experiments were performed in triplicate.

### 2.6 Bacterial growth curves

*E. tarda* cultures (2.0 log CFU/mL in TSB) were placed in 96-well microtiter plates containing 0, 0.1, 0.2, 0.5, 1 and 2% Aq (v/v). The inoculum was calculated by plating onto TSB agar plates at 28 °C for 24 h. Growth was monitored by measuring optical density at 600 nm was monitored at 2 h intervals 20 h using an automatic plate reader (FLUOstar Omega, BMG Labtech, U.K.).

### 2.7 Transepithelial electrical resistance measurements

RTgill-W1 cells were plated onto transwells (1 × 10^5^ cells) (6.5 mm diameter; 0.4 μm pore size; Corning) and grown until apical junctional complexes developed. Transwells were infected with *E. tarda*. TEER was measured 3 days post-infection using an EVOM X meter connected to an Endohm chamber (World Precision Instruments). The presented results are representative of at least three independent experiments.

### 2.8 Proinflammatory and tight junction qPCR analysis

The proinflammatory genes *IL-8*, *IL-1β*, *TNF-α* and barrier integrity genes *cldn-10e*, *cldn-30*, *zo-1* were investigated by qPCR. The RNeasy^®^Plus Mini Kit (Qiagen, United Kingdom) was used to extract the RNA from RTgill-W1 cells and reverse transcribed using Transcriptor First Strand cDNA Synthesis Kit (Roche, United Kingdom). The extracted mRNA was quantified by RT-PCR using QuantiNovaSYBR^®^ Green PCR Kit (Qiagen, United Kingdom) on a LightCycler^®^ 96 (Roche, United Kingdom). Primers (Invitrogen, United Kingdom) are illustrated in **Table 1**. We used the *EF-1α* gene and *β-actin* genes as the internal reference genes for RTgill-W1 cells. We calculated target gene mRNA levels using the 2^-ΔΔCt^ method. The PCR conditions used were as previously described (Valdebenito et al., 2025): initial activation 95 °C for 10 min, 40 cycles of 30 s denaturation at 95 °C, 30s annealing at 60 °C, and 30 s elongation at 72 °C. For the *cldn-10e*, *cldn-30*, *zo-1* gene expression, the PCR conditions were as previously described: heating to 95 °C for 2 min; 45 cycles of 95 °C for 30s and 60 °C for 60s (Trubitt et al., 2015).

**Table 1:** Primers used in this study.

Gene name	Primers	GenBank Code
*IL-8*	F - TGTCGCTGCATTGAGACGGAAA R - AGCGCTGACATCCAGACAAATC	NM_001140710
*IL-1β*	F - GTCACATTGCCAACCTCATCATCG R - GTTGAGCAGGTCCTTGTCCTTGAA	XM_014170479.2
*TNF-α*	F - GTGGAGTGGACAGATGACG R - CTGGTTGTTAGGGTGCTTGG	DQ787157.1
*cldn-10e*	F - ATCAAGGTGGCCTGGTACTG R - GACCAGAGCACAGGGAAGTC	BK006391
*cldn-30*	F - TGATCATTGGAGGAGGGTTC R - AACATAGTCCCTGGGTGCTG	BK006405
*zo-1*	F - AGGCTGTGCTGTTCCTCCTA R - TCCGACGGTAAACATCCTTC	HQ656020
*β-actin*	F - TCCTCGGTATGGAGTCTTGC R - AGCACTGTGTTGGCGTACAG	AF254414
*EF-1α*	F -GCTTACAAAATCGGCGGTAT R - CTTGACGGACACGTTCTTGA	NM001123629.1
*mrp1*	F – TCTGATCCGTTCTGGGATTGG R – CAGGGTATCCAAACCAGCAGT	XM_051888049
*oatp1d1*	F – TGTCATGCCTCTTGGCTTGT R – CCGTCTGTATGAGAGCCAGG	NM_001348086
*sec61a1*	F – GTCACGAGTGCTGTTTTGGG R – TAGAAACGCTTTGCCACTCCT	XM_051898264

### 2.9 Cellular glutathione

The relative concentrations of intracellular glutathione (GSH) were determined by using the GSH/GSSG ratio assay kit (abcam 138881), following the manufacturer’s instructions and as previously described (de Souza et al., 2022). Briefly, infected or un-infected RTgill-W1 cells exposed to 0.1%, 0.2%, 0.5%, and 1% Aq were used to measure cellular glutathione (GSH) concentration. Infected or un-infected RTgill-W1 cells, with or without Aq. After 72 h, cells were collected and washed with cold 1× PBS and 0.5% NP-40 in PBS. Then, we centrifuged the samples and collected the supernatant. We used the deproteinization protocol to remove enzymes. We mixed samples with thiol green solution (1:50), and after 30 min, measured fluorescence (490/520 nm). The GSH transporter mRNA levels (mrp1, oatpd1 and sec61a1) were analysed as previously described and the primers used are included in **Table 1** (Zeng et al., 2025b).

### 2.10 NF-κB activation assay

The activation assay was done as previously indicated (Bundurus et al., 2023). Briefly, NF-κB p65 activation levels were measured in infected or un-infected RTgill-W1 cells after 72 h of infection and exposure to increasing concentrations of Aq (0%, 0.1%, 0.2%, 0.5%, 1% and 2%). Cellular proteins were extracted using a Nuclear Extraction kit from Abcam (ab113474, London, UK), and NF-κB p65 activation in the supernatants was measured using an NF-κB p65 Transcription Factor Assay Kit (ab133112, Colorimetric) according to the manufacturer’s instructions. NF-κB p65 activation was expressed as the ratio of measured absorbance (OD 450 nm) to total nuclear protein (1 mg). Colourimetric changes were measured by absorbance at 550 nm and read on a FLUOstar Omega plate reader (Premier Scientific, Belfast, UK).

### 2.11 Statistical analysis

Statistical analyses were performed using GraphPad software, version 11. In some cases, data were presented as mean ± SD. P-values < 0.05 were considered statistically significant, with significance assessed using Šídák’s test for multiple comparisons. We also used one-way ANOVA for statistical group comparisons where necessary.

## 3 Results

### 3.1 The impact of Aq on E. tarda virulence in RTgill-W1 cells

To assess the effect of Aq on *E. tarda* virulence, we first determined the MIC and MBC to identify a sub-inhibitory concentration suitable for further molecular studies. Based on growth curve and inhibition analyses, our results indicate an MIC of 1% and an MBC of 2% (**Figure 1A**). We excluded the MBC concentration of 2% from mechanistic investigations except for the viability test. A concentration of 0.5% Aq can be considered sub-inhibitory and used as necessary. The results in **Figure 1** suggest that AuraAqua (Aq) can modulate the interaction between *E. tarda* and RTgill-W1 cells. Aq treatment (0%, 0.1%, 0.2%, 0.5%, 1% and 2%) reduced both total bacterial adhesion (**Figure 1B**) and intracellular bacterial (**Figure 1C**) recovery (*P* < 0.0001), indicating that the organic acid-based mixture may interfere with early host–pathogen interactions, as well as with bacterial entry or survival within the epithelial cell model. This effect is relevant because adhesion to host surfaces is an important first step in bacterial colonisation, while internalisation can contribute to persistence and disease progression. These findings suggest that Aq protects RTgill-W1 cells against *E. tarda* infection, potentially by interfering with bacterial adhesion and invasion. However, further work is required to determine whether this effect is mediated primarily through direct effects on the pathogen, changes in host-cell surface properties, or modulation of host immune and barrier responses.

**Figure 1:**
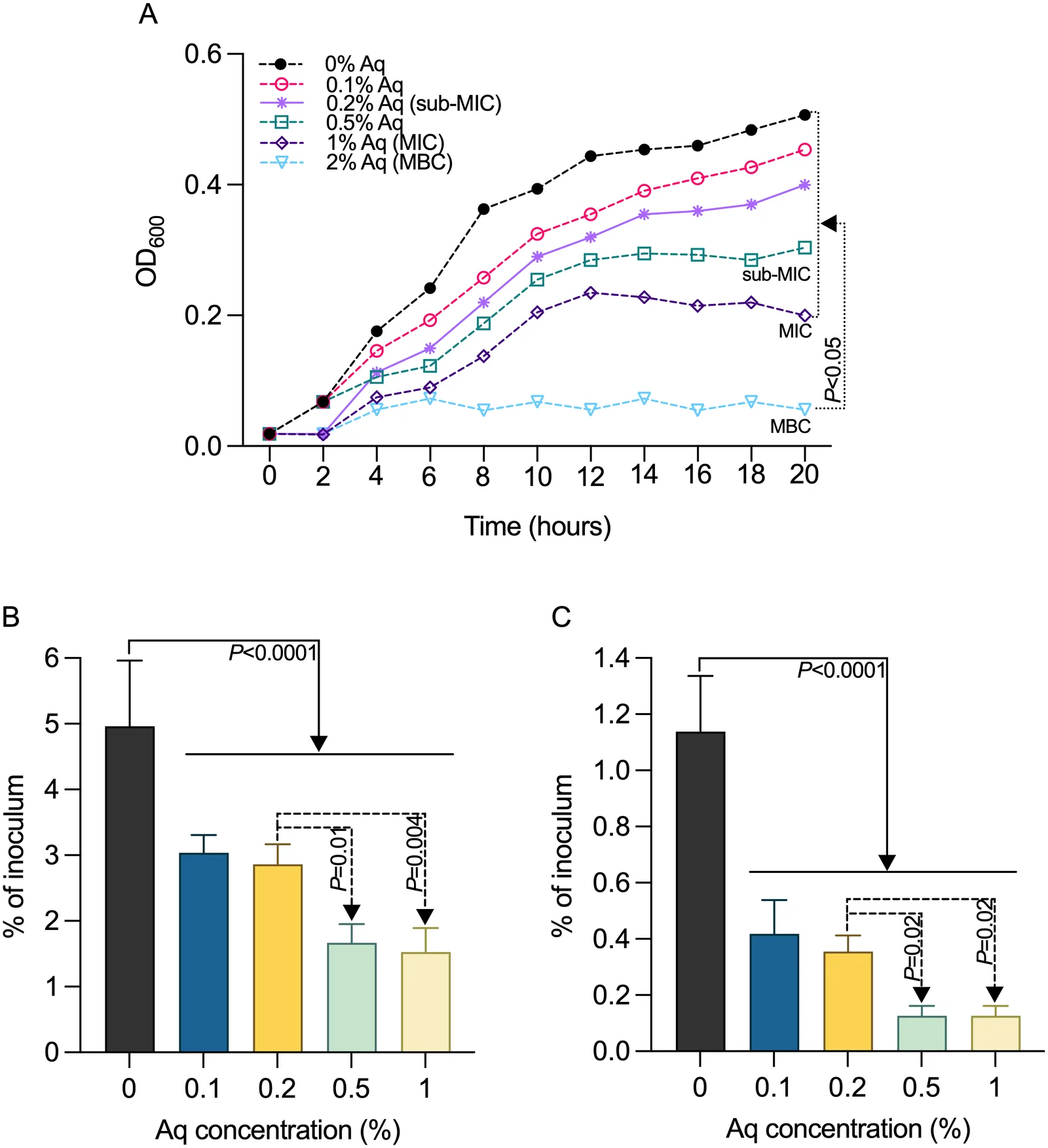
Effect of AuraAqua (Aq) on RTgill-W1 *E. tarda* infection outcomes. **(A)** Growth curve, MIC and MBC representation; **(B)** Total adhesion; **(C)** Internalised bacteria. Data are shown as mean ± SD from three independent experiments. For multiple comparisons the statistical significance was assessed using one-way ANOVA and Šídák’s tests. Significant *P* values are indicated on the graph.

### 3.2 The effect of Aq on infected and un-infected RTgill-W1 cell viability, cytotoxicity and transepithelial resistance

Next, we investigated whether Aq induces cytotoxic effects on RTgill-W1 cells with or without *E. tarda* infection. **Figure 2** shows the effects of AuraAqua (Aq) on RTgill-W1 cell viability, cytotoxicity, and epithelial barrier integrity in infected and un-infected cells. Aq treatment did not markedly reduce cell viability at the concentrations tested, indicating that the mixture was well tolerated by RTgill-W1 cells under these experimental conditions (**Figure 2A**). In *E. tarda*-infected (**Figure 2B**) and un-infected (**Figure 2C**) cells, Aq exposure was associated with reduced LDH release compared with untreated infected controls, suggesting decreased infection-associated cytotoxicity. TEER measurements further indicated that Aq helped preserve epithelial barrier integrity during infection (**Figure 2D**), whereas un-infected monolayers (**Figure 2E**) showed limited disruption. Together, these results suggest that Aq is not directly cytotoxic to RTgill-W1 cells at the concentrations tested and may protect against *E. tarda*-induced cellular damage and barrier dysfunction. The similar LDH-release trends in infected and uninfected cells across different Aq concentrations suggest that the treatment-related effect on membrane integrity was broadly comparable in both conditions, rather than exclusively associated with infection.

**Figure 2:**
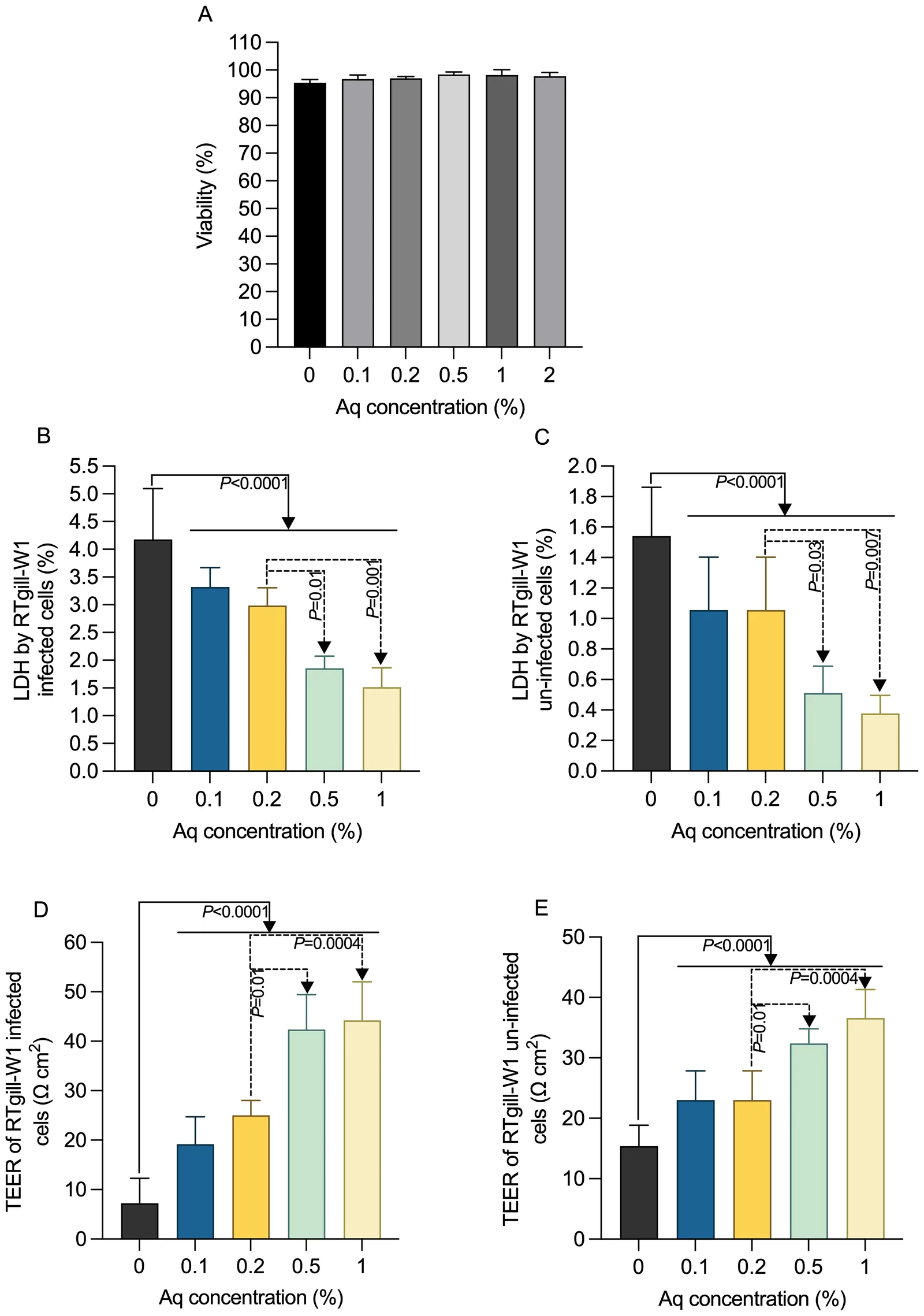
Effect of Aq on RTgill-W1 cell viability and cytotoxicity with or without *E. tarda* infection. RTgill-W1 cells were treated with increasing concentrations (0%, 0.1%, 0.2%, 0.5%, 1% and 2%) of Aq and assessed for cell viability **(A)**, LDH release by infected **(B)** and un-infected **(C)** Aq-exposed cells. TEER values in infected **(D)** and un-infected **(E)** cells in the presence of Aq. Data are shown as mean ± SD from three independent experiments. For multiple comparisons the statistical significance was assessed using one-way ANOVA and Šídák’s tests. Significant *P* values are indicated on the graph.

### 3.3 Aq upregulates genes related to the integrity of RTgill-W1 cell tight junctions

To determine whether Aq supports epithelial barrier integrity at the molecular level, we next examined the expression of tight-junction-associated genes in RTgill-W1 cells with or without *E. tarda* infection (**Figure 3**). Aq treatment increased the relative mRNA expression of *cldn-10e*, *cldn-30*, and *zo-1* in both infected and un-infected cells, indicating that Aq promotes the expression of genes associated with tight-junction structure and epithelial barrier maintenance. This response was particularly relevant during *E. tarda* infection, where increased tight-junction gene expression may contribute to the preservation of epithelial integrity observed in the TEER assay. Together, these findings suggest that Aq enhances tight-junction-associated barrier responses in RTgill-W1 cells and may help protect the gill epithelial monolayer during bacterial infection.

**Figure 3:**
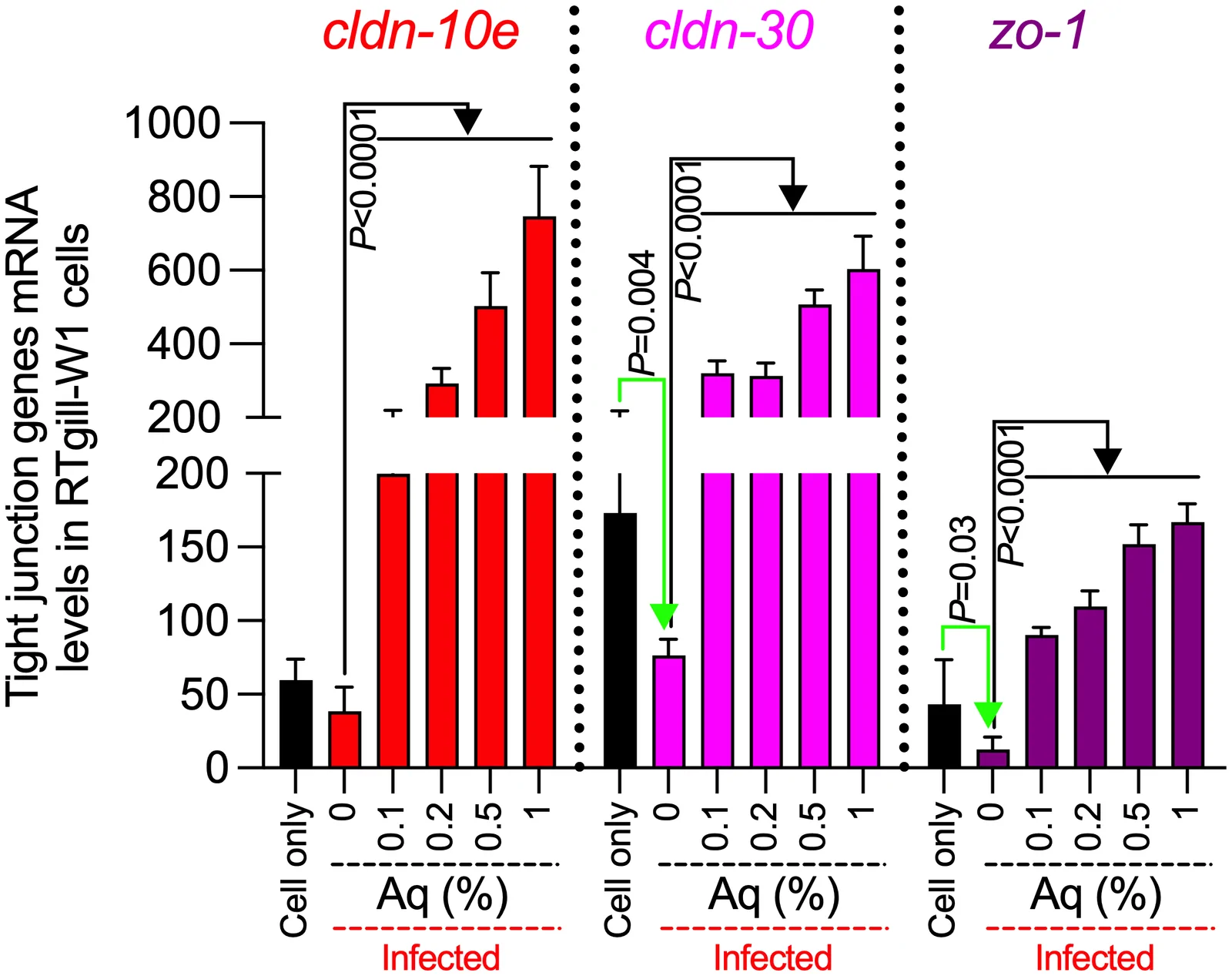
Effect of Aq on tight-junction gene expression in RTgill-W1 cells with or without *E. tarda* infection. RTgill-W1 cells were infected with *E. tarda* or left un-infected and treated with increasing concentrations of Aq (0%, 0.1%, 0.2%, 0.5%, 1% and 2%), as indicated. Relative mRNA expression levels of *cldn-10e*, *cldn-30*, and *zo-1* were determined by qPCR. Data are shown as mean ± SD from three independent experiments. For multiple comparisons the statistical significance was assessed using one-way ANOVA and Šídák’s tests. Significant *P* values are indicated on the graph.

### 3.4 The impact of Aq treatment on RTgill-W1 oxidative stress with or without E. tarda infection

The oxidative stress-related responses of *E. tarda*-infected or un-infected RTgill-W1 cells in the presence of increasing concentrations of Aq (0%, 0.1%, 0.2%, 0.5%, 1% and 2%) were next investigated (**Figure 4**). In un-infected cells, Aq treatment produced no marked concentration-dependent change in CAT activity (**Figure 4A**), while infected cells showed decreased CAT activity following Aq exposure (**Figure 4B**). Baseline SOD activity was detected in the un-infected cells in the presence of Aq (**Figure 4C**). The presence of Aq significantly reduced SOD activity (*P* < 0.0001) in *E. tarda*-infected cells (**Figure 4D**). Baseline H_2_O_2_ levels were detected in the supernatants of un-infected RTgill-W1 cells after Aq treatment (**Figure 4E**), with significantly reduced (*P* < 0.0001) levels observed in infected cells (**Figure 4F**) in the presence of Aq at all concentrations used. Firstly, these results suggest that Aq alone does not cause an oxidative stress response in RTgill-W1 cells. Secondly, Aq may modulate oxidative stress responses in RTgill-W1 cells during *E. tarda* infection, potentially improving cell viability, reducing cytotoxicity, and improving barrier integrity in assays.

**Figure 4:**
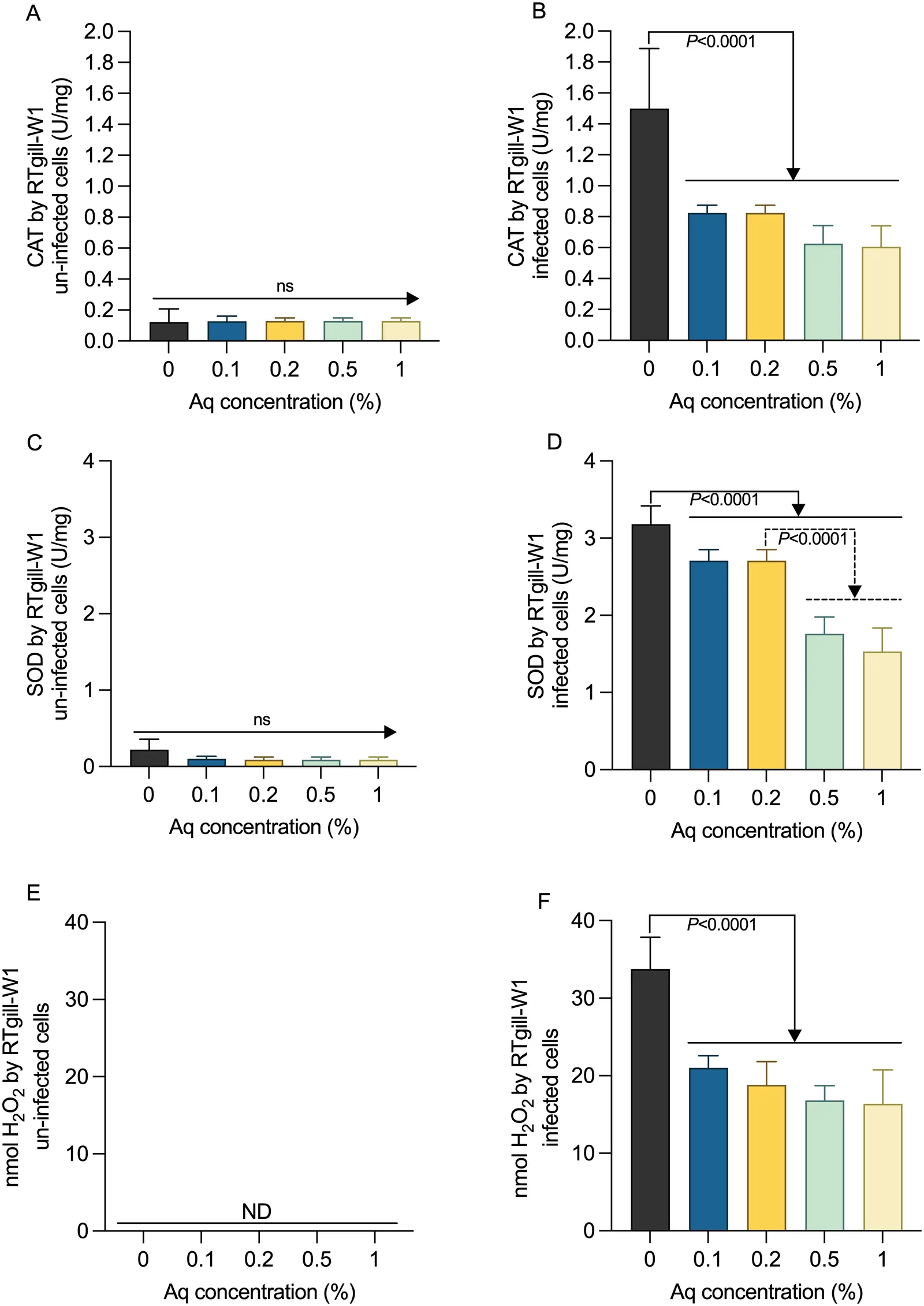
Effect of Aq on CAT, SOD and H_2_O_2_ levels in RTgill-W1 cells only or following 3-day infection with *E. tarda*. **(A)** CAT levels in un-infected cells; **(B)** CAT levels in infected cells; **(C)** SOD levels in un-infected cells; **(D)** SOD levels in infected cells; **(E)** H2O2 levels in un-infected cells; **(F)** H_2_O_2_ levels in infected cells. Cells and/or bacteria were treated with increasing concentrations of Aq (0%, 0.1%, 0.2%, 0.5%, 1% and 2%), as indicated. Data are shown as mean ± SD from three independent experiments. For multiple comparisons the statistical significance was assessed using one-way ANOVA and Šídák’s tests. Significant *P* values are indicated on the graph. ND, not detected.

### 3.5 Effect of Aq on RTgill-W1 cell glutathione and NF-κB activation

We next assessed whether Aq affected intracellular glutathione (GSH) levels and if it is in anyway related to the NF-κB pathway activation in RTgill-W1 cells. **Figure 5** shows that Aq enhances glutathione-dependent antioxidant defence while limiting inflammatory signalling in RTgill-W1 cells. At 72 h post-infection, Aq significantly increased intracellular GSH levels in a dose-dependent manner, with the strongest response in *E. tarda*-infected cells (*P* < 0.0001; **Figure 5A**). A similar, though less pronounced, increase was observed in uninfected cells. In contrast, Aq reduced NF-κB p65 activation, particularly during infection, indicating suppression of infection-induced inflammatory signalling. GSH-transporter mRNA expression was also assessed to examine the molecular mechanisms underlying altered intracellular GSH levels (**Figure 5B**). At the sub-inhibitory concentration of 0.5%, Aq produced a time-dependent increase in GSH without a corresponding increase in H_2_O_2_ production (**Figure 5C**). These findings indicate that Aq-induced oxidative stress did not cause the elevated GSH response. Collectively, the results suggest that Aq strengthens cellular antioxidant capacity while attenuating NF-κB-mediated inflammatory activation during *E. tarda* infection.

**Figure 5:**
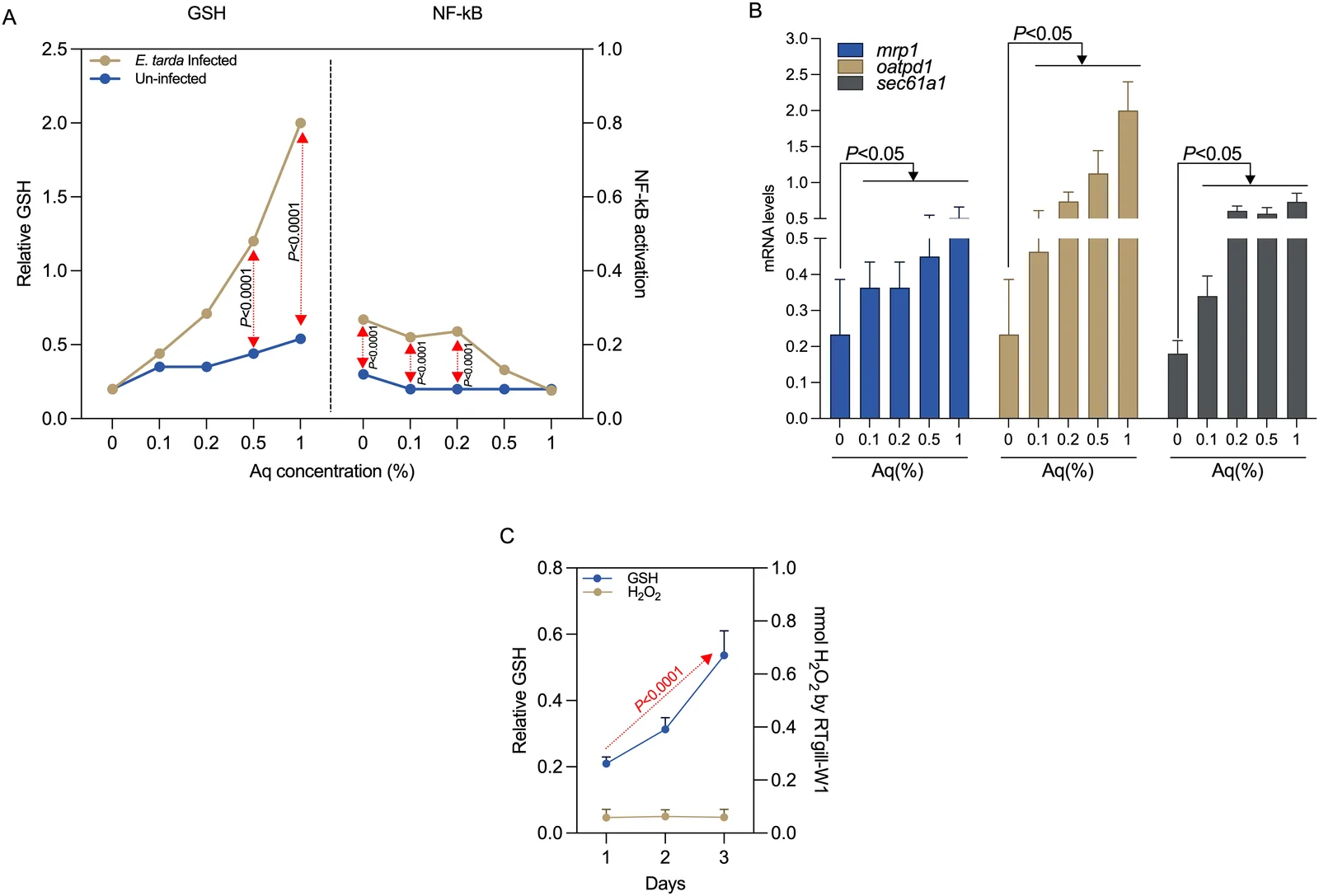
Effect of Aq on intracellular GSH and NF-κB activation in RTgill-W1 cells at 72 h post-infection **(A)**; GSH transporter mRNA levels **(B)** and the time course GSH and H_2_O_2_ levels in RTgill-W1 cells only at 0.5% Aq **(C)**. For multiple comparisons, we assessed statistical significance using one-way ANOVA and Šídák’s test. Significant *P* values are indicated on the graph.

### 3.6 Effect of Aq on IL-8, IL-1β and TNF-β inflammatory gene expression in RTgill-W1 cells

We next investigated whether AuraAqua (Aq) modulates the inflammatory response of RTgill-W1 cells during *E. tarda* infection by measuring IL-8, IL-1β, and TNF-α mRNA expression (**Figure 6**). *E. tarda* infection altered the expression of these inflammatory markers compared with un-infected control cells. Treatment with Aq altered this response in a concentration-dependent manner, with significantly reduced (*P* < 0.0001) IL-8, IL-1β, and TNF-α expression observed at all tested concentrations. These results suggest that Aq may modulate the inflammatory response of RTgill-W1 cells during *E. tarda* infection, potentially contributing to its protective effects on cell viability, cytotoxicity, oxidative stress, and epithelial barrier integrity.

**Figure 6:**
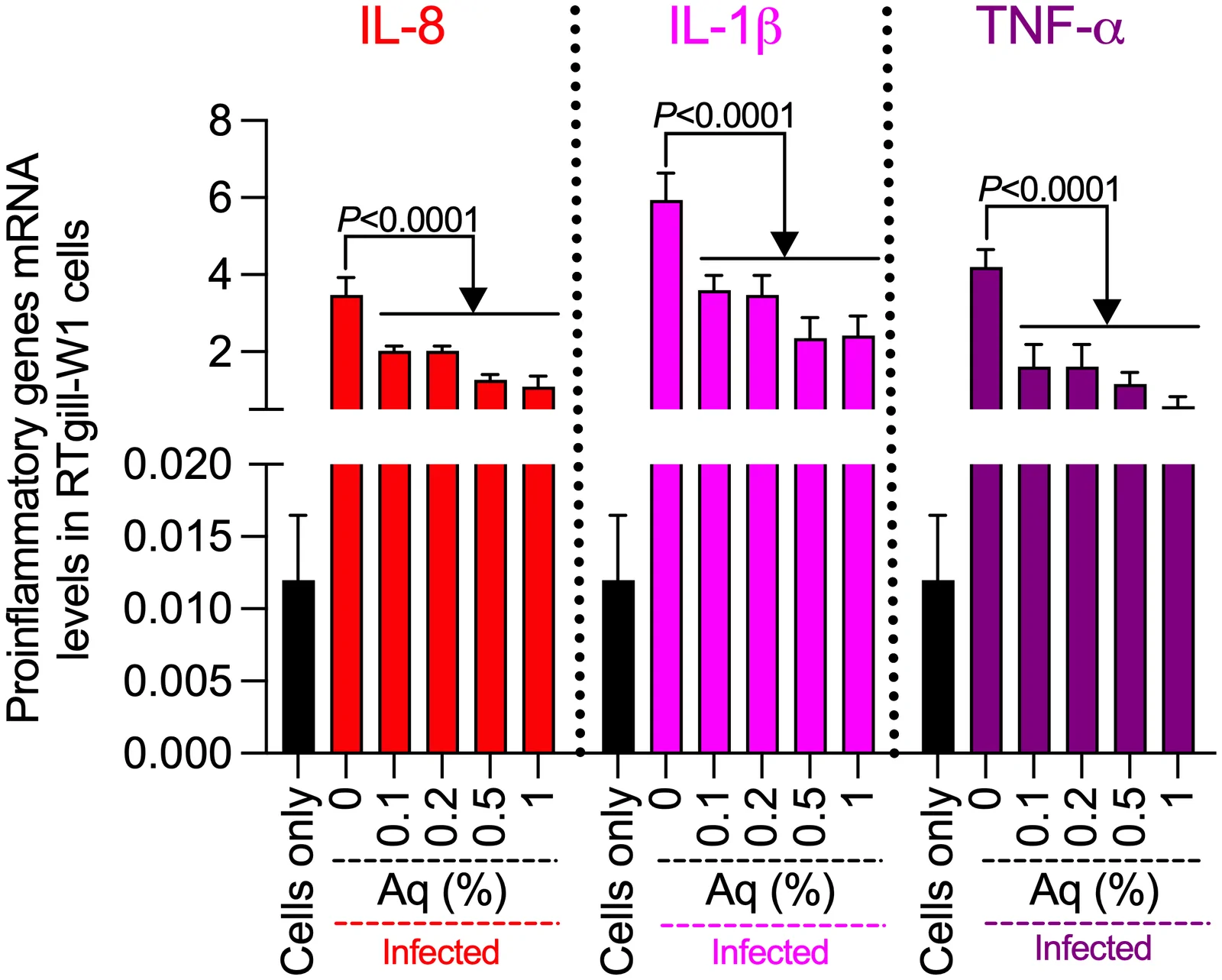
Effect of AuraAqua (Aq) on IL-8, IL-1β and TNF-α inflammatory gene expression in *E. tarda* infected or un-infected RTgill-W1 cells. RTgill-W1 cells were infected with *E. tarda* and treated with increasing concentrations of Aq (0%, 0.1%, 0.2%, 0.5%, 1% and 2%), as indicated. We determined relative mRNA expression levels of IL-8, IL-1β, and TNF-α by qPCR and normalised them to EF-1α. Data are shown as mean ± SD from three independent experiments. For multiple comparisons, the statistical significance was assessed using one-way ANOVA and Šídák’s test. Significant *P* values are indicated on the graph.

## 4 Discussion

The rainbow trout gill cell line (RTgill-W1) has long been used to investigate the role of the gill epithelium during viral (Misk et al., 2022) and bacterial infection (Jawdhari et al., 2025). In this study, initial virulence assays showed that Aq reduced key early events in *E. tarda* infection of RTgill-W1 cells. Aq decreased total bacterial adhesion and intracellular bacterial recovery, indicating impaired attachment, entry, or intracellular survival. Because adhesion and internalisation are central to colonisation and disease progression (Ribet and Cossart, 2015), these findings suggest that Aq attenuates *E. tarda* virulence at the gill epithelial interface, possibly through direct antimicrobial pressure or modulation of host-cell defence responses.

The efficiency of lactate dehydrogenase (LDH) (Zou et al., 2013) and the MTT (3-[4,5-dimethylthiazol-2-yl]-2,5-diphenyltetrazolium bromide) (Fotakis and Timbrell, 2006) assays in estimating cytotoxicity in RTgill-W1 gill cells has previously been demonstrated, with the latter being the most sensitive. In the present study, the MTT assay showed that Aq did not markedly compromise RTgill-W1 cell viability at the concentrations tested, indicating that the organic acid mixture was well tolerated by the gill epithelial cell model. In parallel, LDH release, which reflects loss of membrane integrity and cytotoxic damage (Kumar et al., 2018), was lower in *E. tarda*-infected cells exposed to Aq than in infected untreated controls. Transepithelial electrical resistance (TEER) is a quantitative method for assessing tight junction integrity in cell culture models (Srinivasan et al., 2015). TEER was previously used as a useful model for *in vitro* study of *in vivo* gill physiology in RTgill-W1 cell layers (Trubitt et al., 2015). In this study, TEER measurements showed that Aq helped preserve RTgill-W1 epithelial barrier integrity during *E. tarda* infection. Because TEER reflects tight junction function and monolayer permeability, the infection-associated reduction in resistance indicates disruption of epithelial barrier stability. Aq treatment helped maintain TEER in infected monolayers, suggesting reduced damage to cell–cell junctions and preservation of barrier function. This finding aligns with reduced LDH release and maintained cell viability in Aq-treated infected cells. In contrast, un-infected monolayers exposed to Aq showed limited TEER disruption, indicating that Aq was not intrinsically damaging to the epithelial barrier. In addition to TEER, the cellular junction proteins claudin 10e (*Cldn-10e*), *Cldn-30*, and zonula occludens-1 (*zo-1*) are key to modulating and maintaining the physiological integrity of RTgill-W1 gill cells (Trubitt et al., 2015). Consistent with the TEER results, Aq increased *cldn-10e*, *cldn-30*, and *zo-1* expression in infected and un-infected RTgill-W1 cells, suggesting molecular reinforcement of tight-junction integrity and indicating that Aq may promote barrier-stabilising responses in RTgill-W1 cells during bacterial challenge.

In our study, the organic acid mixture (Aq) increased GSH production in both un-infected and infected RTgill-W1 cells, with concentrations significantly elevated in the latter. Natural extracts were previously shown to stimulate intracellular glutathione (GSH) production, thereby enhancing cellular antioxidant responses by neutralising free radicals (Di Giacomo et al., 2023). A possible mechanism leading to this increase in GSH production might include the upregulation of the NRF2 pathway (Nuclear factor erythroid 2-related factor 2), which is the cellular defence mechanism acting as a cellular regulator of protective, antioxidant, and detoxification genes to maintain cellular homeostasis (Baird and Yamamoto, 2020). Citric acid, a component of Aq, was shown to prevent the depletion of reduced glutathione and to boost the activity of glutathione peroxidase in tissues such as the liver and brain (Abdel-Salam et al., 2014). Dietary malate, another component of Aq, was also shown to increase total antioxidant capacity and the activity of antioxidant enzymes such as glutathione peroxidase (GSH-Px), which work alongside GSH to neutralise harmful free radicals (Zhang et al., 2021). Aq also contains maltodextrin, a polysaccharide that indirectly influences GSH by supporting the amino acids required for GSH synthesis and stimulating pathways that lower oxidative stress (Pourghassem Gargari et al., 2013). The gills serve as a mucosal barrier, and when GSH levels decline, microbial communities on the gills become dysbiotic, allowing pathobionts (disease-causing bacteria) to proliferate at the expense of beneficial microbes (Tongsri et al., 2023). The gills require high levels of GSH to kill invading bacteria and neutralise excess ROS, thereby protecting their own cells from collateral damage (Bandeira Junior and Baldisserotto, 2021).

Although GSH is a major antioxidant that protects cells against oxidative stress, intracellular GSH levels can vary for reasons unrelated to cellular damage. Healthy cells may actively increase GSH reserves as a preventive defence buffer or to support essential metabolic processes (Forman et al., 2009). This can explain the disconnect between the reduced H_2_O_2_ levels observed in both infected and un-infected RTgill-W1 cells, even though the GSH levels were elevated. We also show that the increase in GSH production is not due to Aq-induced oxidative stress, strengthening the claim that Aq is non-cytotoxic. The lowering effect of Aq on CAT and SOD in *E. tarda*-infected RTgill-W1 cells observed in this study is not directly linked to GSH levels observed, since GSH does not directly trigger or regulate the production of CAT and SOD.

The relationship between glutathione (GSH) and pro-inflammatory cytokines in fish gills is a critical axis of the mucosal immune system and the stress response. GSH serves as a primary endogenous antioxidant, while pro-inflammatory cytokines (such as TNF-α and IL-1β) mediate immune and inflammatory responses (Song et al., 2025). In our study, Aq was shown to induce in both *E. tarda*-infected and un-infected RTgill-W1 cells increased relative GSH levels, reduced H_2_O_2_ production and lower mRNA levels of IL-8, IL-1β and TNF-α. All these events were associated with reduced activation of the NF-κB pathway. These results are supported by previous findings indicating that reduced GSH and increased oxidative stress activate the NF-κB pathway (Shi et al., 2015), triggering the transcription and release of pro-inflammatory cytokines such as TNF-α, IL-1β, and IL-8 in gill tissue (Kwon et al., 2019). Organic acids in a mixture can also block activation of the NF-kB pathway during infection, reducing oxidative stress and the proinflammatory response (Bundurus et al., 2023). The results from this RTgill-W1 gill cell infection model could potentially predict *in vivo* outcomes in fish, since this model has previously been indicated as suitable for evaluating gill function and pathogenicity in fish (Lee et al., 2025).

## 5 Conclusions

In conclusion, this study demonstrates that AuraAqua (Aq), an organic acid-based antimicrobial mixture, protects RTgill-W1 gill epithelial cells from *Edwardsiella tarda*-induced cellular injury (**Figure 7**). Aq reduced bacterial adhesion and intracellular bacterial recovery, preserved cell viability, lowered LDH release, and helped maintain epithelial barrier integrity, indicating protection against infection-associated cytotoxicity and barrier disruption. These effects were accompanied by increased expression of tight-junction-associated genes, enhanced intracellular glutathione levels, reduced oxidative stress responses, decreased NF-κB activation, and downregulated expression of pro-inflammatory markers, including IL-8, IL-1β, and TNF-α. Together, these findings suggest that Aq supports gill epithelial resilience by limiting early host–pathogen interactions, strengthening antioxidant defences, and suppressing inflammatory signalling. The results highlight the potential of organic acid-based antimicrobial mixtures as functional strategies to reduce pathogen-associated gill damage in aquaculture-relevant models. Further *in vivo* studies are warranted to confirm these effects in fish and to clarify the mechanisms underlying Aq-mediated modulation of the epithelial barrier, oxidative stress, and immune responses.

**Figure 7:**
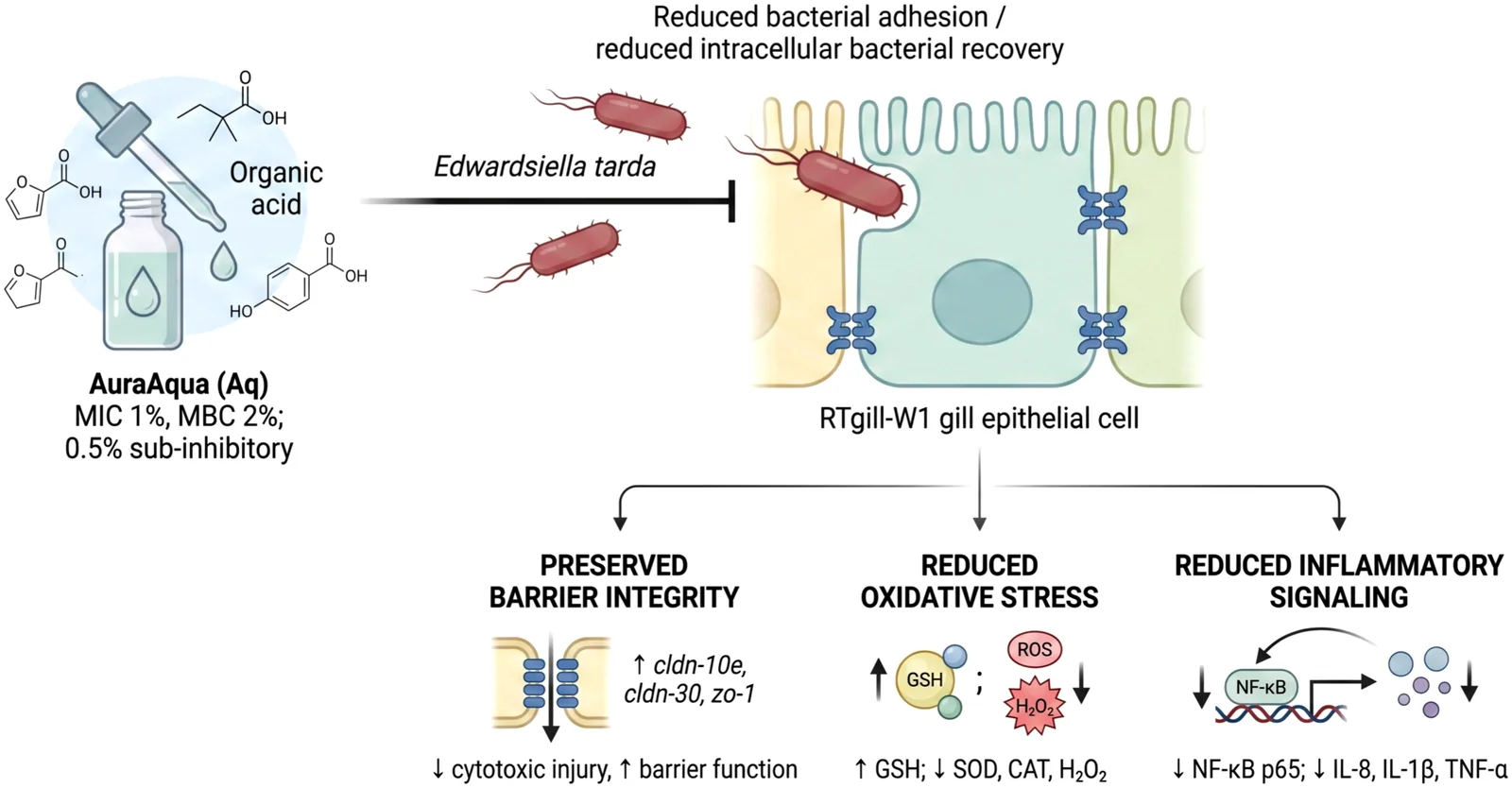
Proposed model summarising the protective effects of AuraAqua (Aq), an organic acid-based antimicrobial mixture, in RTgill-W1 gill epithelial cells during *Edwardsiella tarda* infection. Aq reduces early host–pathogen interactions by limiting bacterial adhesion and intracellular bacterial recovery, while preserving cell viability, lowering LDH release, and maintaining epithelial barrier integrity. These effects are associated with increased tight-junction gene expression, enhanced intracellular glutathione (GSH) levels, reduced H_2_O_2_ accumulation, decreased CAT and SOD responses during infection, reduced NF-κB p65 activation, and downregulation of IL-8, IL-1β, and TNF-α expression. Together, these responses suggest that Aq supports gill epithelial resilience by strengthening antioxidant defence, stabilising barrier function, and suppressing inflammatory signalling. Created in Biorender. Biorender.com/RE2A4K9C73.

## Data Availability

The raw data supporting the conclusions of this article will be made available by the authors, without undue reservation.
